# Conditional Information-Bottleneck Graph Clustering for Structured Representation Learning in Dynamic Vehicular ISAC Networks

**DOI:** 10.3390/e28080884

**Published:** 2026-08-05

**Authors:** Yiyang Wu, Hongqiu Zhu

**Affiliations:** 1 School of Electrical Engineering and Telecommunications, The University of New South Wales, Sydney, NSW 2052, Australia; 2 School of Automation, Central South University, Changsha 410083, China; hqcsu@csu.edu.cn

**Keywords:** conditional information bottleneck, information-theoretic graph clustering, structured representation learning, integrated sensing and communication, multi-agent resource optimization

## Abstract

Dynamic vehicular integrated sensing and communication (ISAC) requires representations that remain compact, decision-relevant, and structurally stable as mobility rewires interference and sensing relations. This paper presents IC-GMRO, a conditional information-bottleneck graph-clustering framework for structured representation learning in multi-agent resource optimization. At each control epoch, vehicles, roadside units, targets, and typed interactions form a temporal heterogeneous graph. A context-conditioned variational bottleneck suppresses nuisance variation while retaining action-relevant information; balanced soft graph clusters then convert the latent space into reusable coordination codes. Feasibility-masked policies jointly select association, beam, resource block, transmit power, and sensing-time ratio. The analysis distinguishes representation-level information guarantees from the idealized potential and projected-dual arguments used only to motivate the practical neural updates. Controlled simulations and component ablations show improved utility, sensing success, latency robustness, and cross-density robustness relative to greedy, flat, and graph-only baselines.

## 1. Introduction

Vehicular integrated sensing and communication (ISAC) networks must turn a rapidly changing physical scene into resource decisions within a short control interval. A vehicle may transmit safety data, illuminate a target, estimate range or angle, and exchange sensor products while sharing spectrum, power, beams, and edge-computing capacity. The resulting problem jointly couples communication quality, sensing geometry, interference, queueing, and reliability; it cannot be reduced to a conventional scheduler or a standalone radar planner [1,2,3]. Resource-allocation formulations that explicitly couple sensing and communications reinforce this systems view [4].

Recent learning-based work shows that reinforcement learning and learning-based optimization can coordinate communication, sensing, and computing resources in mobile systems. Representative studies include vehicular ISCC allocation, Internet-of-Vehicles ISAC control, and deep learning-based uplink ISAC design [5,6,7]. Dynamic graph learning has also been used to model nonstationary relational observations [8]. Complementary ISAC studies address coverage-rate tradeoffs, aerial assistance, and NOMA-enabled integration [9,10,11]. However, many controllers still represent the environment through a fixed-length vector or a static neighborhood. Such representations become brittle when vehicles enter or leave a region, sensing visibility changes, or mobility rewires the dominant interference relations.

Graph neural networks supply an appropriate structural inductive bias because relation-specific message passing can be reused across graph sizes and topologies [12,13,14]. Information-theoretic graph contrastive learning provides a complementary account of how graph encoders can retain structural invariants [15]. Relation-aware power control, temporal graph convolution, and graph convolutional encoders demonstrate that structured message passing can be specialized to wireless and temporal data [16,17,18]; attention-based aggregation further permits selective weighting of neighbors [19]. Yet a relational encoder alone does not answer the central representation question: which information should survive compression? In a dynamic vehicular service, short-term fading and exchangeable agent identity can be nuisance variation, whereas density, service class, and safety context remain decision-relevant. The learning problem is therefore explicitly information-theoretic: learn a compact graph-dependent representation that preserves the information needed for constrained resource decisions while discarding irrelevant variation.

The information-bottleneck principle formalizes the tradeoff between compression and predictive relevance [20,21,22]. Information-plane analysis and recent compression reviews clarify how this tradeoff can be interpreted in neural representations [23,24]. Conditional and unique-information inequalities further motivate retaining context that explains a downstream decision while suppressing redundant variability [25]. Related studies on structured temporal graphs and information diffusion motivate adaptive relation modeling under nonstationary observations [26,27,28]; dynamic attention-calibrated graph learning provides a further example of relation adaptation as graph structure evolves [29]. For multi-agent control, the relevance variable need not be a semantic label: it can be a feasible action or a value-relevant decision descriptor. In parallel, graph clustering can convert continuous representations into reusable coordination states by grouping agents that experience comparable interference, sensing, and queueing pressure. In this setting, clustering is not post hoc visualization; it is a structured, decision-coupled representation layer.

This paper develops IC-GMRO, a conditional information-bottleneck graph-clustering framework for joint vehicular ISAC resource optimization. At each control epoch, vehicles, roadside units (RSUs), and targets form a temporal heterogeneous graph with typed interference, association, and sensing-visibility edges. A relation-aware encoder produces node embeddings; a context-conditioned variational bottleneck forms compact latent codes; and balanced soft graph clusters turn those codes into reusable coordination context. Shared decentralized actors then use feasibility masks to select an RSU, radar beam, communication resource block, transmit power, and sensing-time ratio, while centralized critics and dual variables are used during training to account for joint utility and average constraints.

The contribution is intentionally framed around information-theoretic structured representation learning rather than a generic GNN–MARL pipeline. First, we formulate the allocation problem as a graph-structured constrained Markov game that couples communication throughput, sensing information, latency, energy, interference, and reliability. Second, we introduce a conditional information-bottleneck objective in which density and service class are retained as legitimate conditioning variables, and combine it with balanced soft graph clustering to construct decision-relevant latent codes. We make explicit how confident, balanced assignments increase the usable discrete information carried by the cluster variable. Third, we develop a feasibility-masked centralized-training and decentralized-execution policy for hybrid discrete-continuous resource actions. Fourth, we analyze encoder permutation equivariance, a representation-induced one-step decision-loss bound, an idealized proximal regularized-potential ascent property, and a projected-dual regret inequality. The latter two results concern the specified idealized updates; they are not global convergence or hard-feasibility guarantees for a neural implementation under arbitrary nonstationarity.

Controlled simulations compare IC-GMRO with greedy, flat multi-agent, and graph-only baselines under common mobility, channel, and action settings. In addition to system-level utility, sensing, and latency measures, the ablations are interpreted as evidence for the decision value of conditional compression and information-theoretic clustering. The paper does not claim that its clusters are externally supplied semantic classes or that it is a general-purpose clustering benchmark; the intended contribution is task-coupled structured representation learning for a dynamic control problem.

The remainder of this paper is organized as follows. Section 2 introduces the dynamic vehicular ISAC system and the constrained optimization problem. Section 3 and Section 4 present IC-GMRO, including graph encoding, conditional compression, clustering, and multi-agent learning. Section 5 gives the analytical properties. Section 6 reports controlled simulations and representation-focused interpretation, and Section 7 concludes the paper. The notation used throughout the article is summarized in Table 1.

## 2. Problem Description and Preliminaries

### 2.1. Dynamic Vehicular Sensor Network

Consider an urban control region containing a set of vehicles $V_{t}=\left\{1,\ldots ,N_{t}\right\}$, roadside units (RSUs) $R_{t}$, and sensing targets $T_{t}$ at discrete epoch *t*. A vehicle is equipped with a joint radar-communication front end. The front end transmits a waveform over one of *B* beams and one of *K* orthogonal resource blocks. A short control interval is divided between radar probing and packet transmission. Let the hybrid action of vehicle *i* be

(1) $$a_{i,t}=\left(r_{i,t},b_{i,t},k_{i,t},p_{i,t},\rho_{i,t}\right)\in A_{i}\left(t\right),$$

where $r_{i,t}\in R_{t}$ is an associated RSU, $b_{i,t}\in \left\{1,\ldots ,B\right\}$ is a beam index, $k_{i,t}\in \left\{1,\ldots ,K\right\}$ is a communication block, $p_{i,t}\in \left[0,P_{\max}\right]$ is a power level, and $\rho_{i,t}\in \left[\rho_{\min},\rho_{\max}\right]$ is the sensing ratio. The feasible set incorporates beam availability, RSU association, maximum power, and a local safety mask.

**Figure 1:**
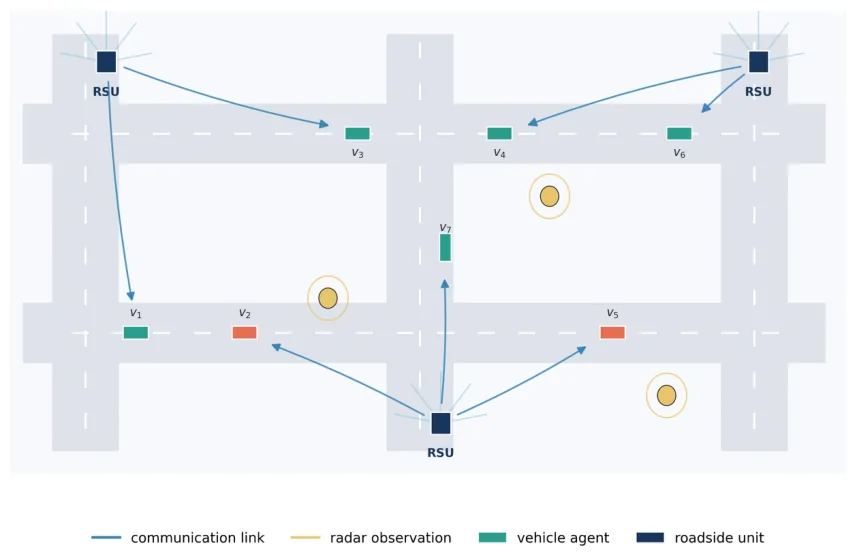
Dynamic vehicular joint radar-communication environment with RSUs, targets, and moving vehicles.

Each vehicle observes a local vector $o_{i,t}$ containing its queue state, velocity, relative geometry, predicted channel quality, residual energy, and a compact radar-quality descriptor. The global state $s_{t}$ includes all vehicle positions, channels, target states, and RSU loads, but it is used only during centralized training. The traffic state changes through vehicle motion, target motion, packet arrivals, and fading. Hence the active node set and edge set can change from one epoch to the next. Figure 1 depicts the coupled sensing, communication, and edge-coordination relations considered in one control region.

### 2.2. Joint Communication and Radar Metrics

Let $h_{ij,t}^{\left(k\right)}$ denote the complex effective channel from vehicle *i* to its associated RSU on block *k*, after beam selection. The instantaneous communication SINR is modeled as

(2) $$\gamma_{i,t}=\frac{p_{i,t}{\left|h_{ir_{i,t},t}^{\left(k_{i,t}\right)}\right|}^{2}}{\sigma_{n}^{2}+\sum_{j\neq i}I\left\{k_{j,t}=k_{i,t}\right\}p_{j,t}{\left|h_{jr_{i,t},t}^{\left(k_{i,t}\right)}\right|}^{2}}.$$

The communication rate under the available transmission fraction is

(3) $$R_{i,t}=\left(1-\rho_{i,t}\right)W_{k_{i,t}}\log_{2}\left(1+\gamma_{i,t}\right),$$

where $W_{k}$ denotes the bandwidth of resource block *k*. The packet delay is

(4) $$D_{i,t}=D_{i,t}^{q}+\frac{L_{i,t}}{R_{i,t}+\epsilon_{R}}+D_{r_{i,t},t}^{\mathrm{proc}},$$

where $D_{i,t}^{q}$ is the queueing delay, $L_{i,t}$ is the packet size, $D_{r,t}^{\mathrm{proc}}$ is RSU processing delay, and $\epsilon_{R}>0$ prevents division by zero. Equation (4) is therefore a packet-service latency and does not include controller computation. For the closed-loop metric reported later, we add the measured online controller time $D_{i,t}^{\mathrm{ctrl}}$, from receipt of the current observation and graph snapshot to production of a feasible action, and define

(5) $$D_{i,t}^{\mathrm{cl}}=D_{i,t}+D_{i,t}^{\mathrm{ctrl}}.$$

The controller term includes graph construction/encoding, cluster-head evaluation, action masking, and actor inference; it excludes training, replay-buffer updates, and centralized-critic optimization.

Radar quality is described through a two-dimensional range-angle Fisher information matrix. Let $\vartheta_{i,t}$ contain the target’s relative range and angle, and let $y_{i,t}$ be the received echo. The radar information matrix is

(6) $$J_{i,t}=E\left[\left(\frac{\partial \log p(y_{i,t}|\vartheta_{i,t})}{\partial \vartheta_{i,t}}\right){\left(\frac{\partial \log p(y_{i,t}|\vartheta_{i,t})}{\partial \vartheta_{i,t}}\right)}^{\;⊤}\right].$$

For a positive definite matrix, the position error bound (PEB) is

(7) $${\mathrm{PEB}}_{i,t}=\sqrt{\mathrm{tr}\left(J_{i,t}^{-1}\right)}.$$

The effective radar SNR scales with the sensing-time ratio, selected beam gain, transmit power, and the target’s radar cross section. Thus $\rho_{i,t}$ creates a direct sensing-communication trade-off rather than acting as an independent control.

### 2.3. Constrained Markov Game

The system is modeled as the following partially observable constrained Markov game:

(8) $$G=\langle V_{t},S,\left\{O_{i}\right\},\left\{A_{i}\right\},P,\left\{u_{i}\right\},\left\{g_{m}\right\}\rangle .$$

Vehicle *i* obtains a shaped local utility

(9) $$\begin{array}{c} u_{i,t}=w_{R}{\bar{R}}_{i,t}+w_{S}{\bar{S}}_{i,t}-w_{D}{\bar{D}}_{i,t}-w_{E}{\bar{E}}_{i,t}-w_{I}{\bar{I}}_{i,t}-w_{O}I\left\{\gamma_{i,t}<\gamma_{\min}\right\}, \end{array}$$

where barred variables are normalized, ${\bar{S}}_{i,t}=-\log\left({\mathrm{PEB}}_{i,t}/{\mathrm{PEB}}_{\mathrm{ref}}\right)$ is a sensing-information score, $E_{i,t}$ is energy use, and $I_{i,t}$ is a measured interference contribution. The joint objective is to maximize the discounted expected return subject to average reliability constraints:

(10) $$\max_{\pi }\;J\left(\pi \right)=E_{\pi }\left[\sum_{t=0}^{T-1}\zeta^{t}\sum_{i\in V_{t}}u_{i,t}\right],\;s.t.\;E_{\pi }\left[g_{m}\left(s_{t},a_{t}\right)\right]\leq 0,\;m\in M.$$

The constraints include deadline violation, radar PEB, maximum power, and packet outage. Because $N_{t}$ and the interference topology vary, a direct tabular state-action construction is impractical. A structured representation is required before policy learning.

**Remark** **1.** *The game couples resources that are often optimized separately. Increasing transmit power can improve the desired communication link and radar echo strength, but it may also raise interference and degrade a neighboring vehicle’s sensing decision. Likewise, assigning more sensing time can reduce the PEB while increasing packet delay. A useful controller therefore requires relational state modeling and constrained long-horizon reasoning, not only pointwise link adaptation.*

## 3. Model and Analysis

### 3.1. Temporal Heterogeneous Graph Construction

At epoch *t*, IC-GMRO constructs a heterogeneous graph $G_{t}=\left(N_{t},E_{t}\right)$ with nodes $N_{t}=V_{t}\cup R_{t}\cup T_{t}$. Vehicle nodes hold local communication, motion, queue, and radar descriptors. RSU nodes hold congestion, beam occupancy, and edge-compute load. Target nodes hold relative position, predicted velocity, and visibility confidence. Three edge families are used: vehicle-to-vehicle interference edges, vehicle-to-RSU association edges, and vehicle-to-target sensing edges. An interference edge i→j is retained when

(11) $$\omega_{ij,t}=\frac{p_{i,t}{\left|h_{ij,t}\right|}^{2}}{\sigma_{n}^{2}+p_{j,t}{\left|h_{jj,t}\right|}^{2}}>\omega_{0}\;\mathrm{or}\;d_{ij,t}<d_{0}.$$

This rule preserves strong interference relations and nearby mobility interactions while keeping the graph sparse.

**Figure 2:**
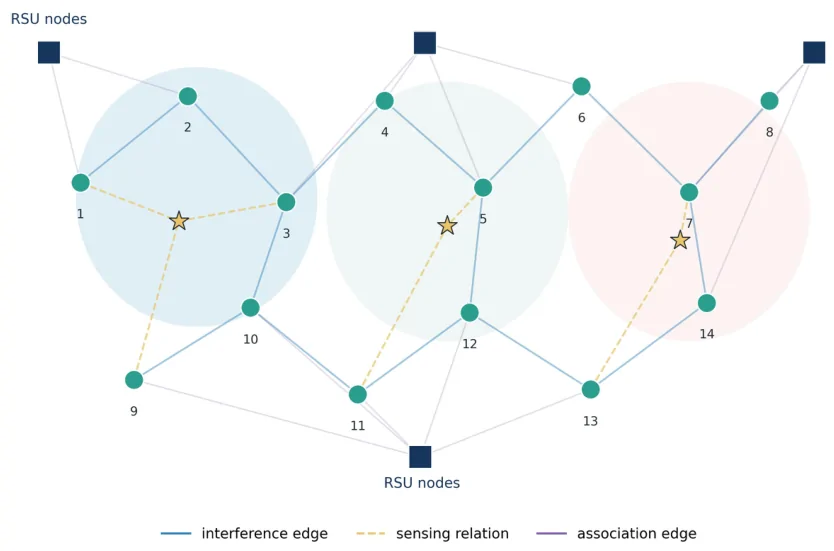
Dynamic heterogeneous graph with interference, sensing, and association relations. Teal circles, navy squares, and yellow stars denote vehicle, RSU, and sensing-target nodes, respectively; solid blue, solid purple, and dashed yellow edges denote interference, association, and sensing relations, while the shaded regions indicate soft graph clusters.

The node feature of vehicle *i* is $x_{i,t}=\left[{\tilde{q}}_{i,t},{\tilde{v}}_{i,t},{\tilde{e}}_{i,t},{\tilde{\gamma }}_{i,t}^{\mathrm{pred}},{\tilde{s}}_{i,t}^{\mathrm{rad}},{\tilde{d}}_{i,t}^{\mathrm{tar}}\right]$. A typed edge feature is $e_{ij,t}=\left[{\tilde{d}}_{ij,t},\tilde{\Delta }v_{ij,t},{\tilde{I}}_{ij,t},{\tilde{\alpha }}_{ij,t},\chi_{ij}\right]$, where $\chi_{ij}$ encodes the relation type. Figure 2 illustrates the resulting graph. Unlike a fixed connectivity graph, it contains both information-flow edges and sensing dependencies.

A relation-aware message-passing layer is defined by

(12) $$h_{i,t}^{\left(\ell +1\right)}=\sigma \left(W_{0}^{\left(\ell \right)}h_{i,t}^{\left(\ell \right)}+\sum_{r\in R_{e}}\sum_{j\in N_{i}^{r}}\alpha_{ij,t}^{r,(\ell )}W_{r}^{\left(\ell \right)}\left[h_{j,t}^{\left(\ell \right)}∥e_{ij,t}^{r}\right]\right),$$

where $R_{e}$ is the edge-type set, ∥ denotes concatenation, and $\alpha_{ij,t}^{r,(\ell )}$ is a normalized attention coefficient:

(13) $$\alpha_{ij,t}^{r,(\ell )}=\frac{\exp\{\psi_{r}\left(h_{i}^{\left(\ell \right)},h_{j}^{\left(\ell \right)},e_{ij}^{r}\right)\}}{\sum_{m\in N_{i}^{r}}\exp\left\{\psi_{r}\left(h_{i}^{\left(\ell \right)},h_{m}^{\left(\ell \right)},e_{im}^{r}\right)\right\}}.$$

The embeddings preserve the distinction between a high-interference neighbor and a target that provides sensing benefit.

### 3.2. Conditional Information Bottleneck and Information-Theoretic Graph Clustering

The graph encoder should retain the information needed to choose a resource action while discarding incidental variation caused by small-scale fading, transient density, or identity of an exchangeable neighbor. Let $X_{i}$ denote the graph-derived context, $D_{i}$ the density–service descriptor, and $A_{i}^{⋆}$ a feasible hybrid action drawn from a lagged centralized teacher policy that observes the training-only global graph and uses the same feasibility masks as the actor. The teacher is warm-started with the constrained critic, updated periodically by an exponential moving average of the current policy, and treated with stop-gradient when generating labels; it is therefore slowly co-adapted rather than optimized through the bottleneck loss. Let $Z_{i}$ denote the learned latent. We seek a representation that minimizes

(14) $$L_{\mathrm{CIB}}=I\left(X_{i};Z_{i}|D_{i}\right)-\beta I\left(Z_{i};A_{i}^{⋆}|D_{i}\right),$$

where the first term compresses conditional nuisance information and the second preserves decision relevance. The conditional form is important: traffic density and service class are not nuisances; they are legitimate operating contexts.

We implement a variational upper bound for the first term and a classification lower bound for the second term:

(15) $$\begin{array}{c} {\hat{L}}_{\mathrm{CIB}}=\frac{1}{\left|B\right|}\sum_{i\in B}D_{\mathrm{KL}}\left(q_{\psi }\left(z_{i}|x_{i},d_{i}\right)∥r_{\nu }\left(z_{i}|d_{i}\right)\right)-\beta \frac{1}{\left|B\right|}\sum_{i\in B}\log p_{\omega }\left(a_{i}^{⋆}|z_{i},d_{i}\right). \end{array}$$

The learned posterior is Gaussian, $q_{\psi }\left(z_{i}|x_{i},d_{i}\right)=N\left(\mu_{i},\mathrm{diag}\left(\sigma_{i}^{2}\right)\right)$, and the context-conditioned prior $r_{\nu }$ prevents unnecessary compression of density information. For the mixed action, the decoder likelihood is factorized into masked categorical factors for $\left(r_{i},b_{i},k_{i}\right)$, a truncated Gaussian factor for $p_{i}$, and a transformed beta factor for $\rho_{i}$; hence $A_{i}^{⋆}$ may be stochastic during training while its feasible mode/mean is used for deterministic evaluation.

To organize latents into reusable coordination groups, we introduce *C* soft graph clusters. Define the clustering input $U_{i}=\left[Z_{i}∥{\bar{Z}}_{N_{i}}\right]$, so that the probabilistic notation matches the neighbor-aware implementation. The assignment of vehicle *i* is

(16) $$q_{i,c}=q_{\theta }\left(C_{i}=c|U_{i},D_{i}\right)={\mathrm{softmax}}_{c}\left(\frac{w_{c}^{⊤}u_{i}}{\tau_{c}}\right),\;c\in \left\{1,\ldots ,C\right\},$$

where ${\bar{z}}_{N_{i}}$ is the attention-weighted neighbor summary and $\tau_{c}$ is a temperature. We use the context-matched cluster regularizer

(17) $$L_{\mathrm{clu}}=\sum_{d\in D_{B}}\frac{|B_{d}|}{\left|B\right|}\;\left[\frac{1}{|B_{d}|}\sum_{i\in B_{d}}H\left(q_{i}\right)+\xi D_{\mathrm{KL}}\;\left(\frac{1}{|B_{d}|}\sum_{i\in B_{d}}q_{i}∥u_{C}\right)\right],$$

where $B_{d}$ is the subset sharing context *d*; rare strata are accumulated in a short replay window before the balance term is evaluated. The first term promotes confident local assignments, whereas the second prevents context-specific collapse into one cluster.

**Proposition** **1** (Information carried by balanced soft clusters)**.** *Let $C_{i}$ be sampled from $q_{\theta }\left(C_{i}|U_{i},D_{i}\right)$ and fix $D_{i}=d$. For a context-matched batch with mean assignment $\bar{q}\left(d\right)$,*

(18) $$I\left(C;U|D=d\right)=H\left(C|D=d\right)-E_{U|d}\;\left[H\left(q_{\theta }\left(C|U,d\right)\right)\right].$$

*Moreover, $H\left(\bar{q}\left(d\right)\right)=\log C-D_{\mathrm{KL}}\left(\bar{q}\left(d\right)∥u_{C}\right)$. Thus, confident assignments and balanced context-marginal use increase the discrete information carried by C about the implemented clustering input U.*

**Proof.**  The first equality is the conditional mutual-information identity. The sample-entropy term in (17) estimates $E_{U|d}\left[H\left(q_{\theta }\left(C|U,d\right)\right)\right]$. For the second statement, substitute the uniform vector $u_{C}$ into the definition of KL divergence. Minimizing the divergence to $u_{C}$ therefore maximizes the entropy of the context-marginal assignment. Taken together, the two terms make the cluster index informative about *U* without prescribing an external semantic label. □

**Figure 3:**
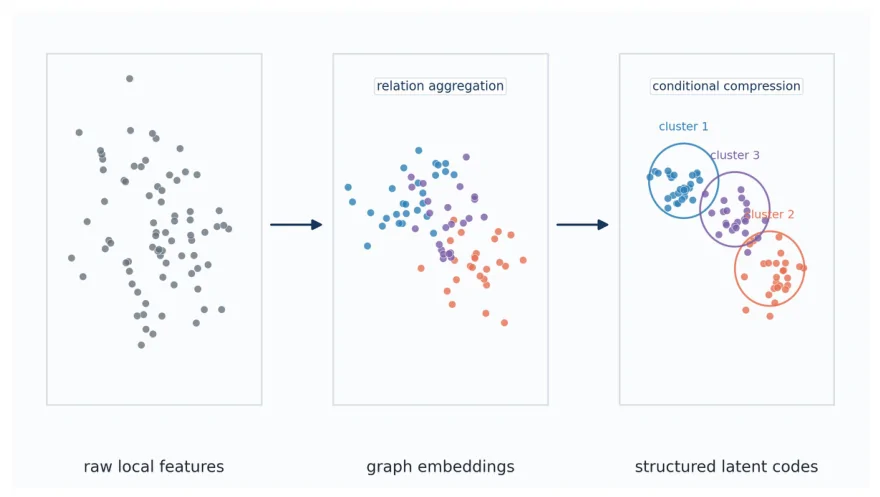
Information-theoretic graph clustering: relation-aware aggregation and conditional compression form compact coordination codes with balanced soft assignments.

This proposition clarifies the information role of clustering in IC-GMRO. It does not, by itself, prove action relevance: that link is supplied by the teacher-decoder term and by the cluster-conditioned actor, and must ultimately be checked with decision and stability diagnostics. The cluster head is not designed to recover a pre-defined taxonomy of vehicles. Figure 3 visualizes the progression from local observations to graph-aware and conditionally compressed coordination codes.

### 3.3. Potential-Shaped Coordination Utility

A pure global reward gives every vehicle the same feedback but provides weak credit assignment. Purely local rewards can create selfish allocations. IC-GMRO uses a potential-shaped utility that retains a global objective while exposing a measurable local marginal contribution. Define the regularized team potential

(19) $$\Phi_{\tau }\left(s_{t},a_{t}\right)=\sum_{i\in V_{t}}u_{i,t}-\kappa \sum_{\left(i,j\right)\in E_{t}^{\mathrm{int}}}{\bar{I}}_{ij,t}+\tau \sum_{i\in V_{t}}H\left(\pi_{i}\left(\cdot |o_{i,t}\right)\right).$$

The first term rewards rate and sensing quality; the second explicitly prices interference; the entropy term supports exploration and yields a smooth best response. For each agent, the counterfactual marginal reward is

(20) $$r_{i,t}=\Phi_{\tau }\left(s_{t},a_{t}\right)-\Phi_{\tau }\left(s_{t},\left(a_{-i,t},a_{i,t}^{\mathrm{ref}}\right)\right),$$

where $a_{i,t}^{\mathrm{ref}}$ is a feasible reference action. This definition rewards the part of the potential attributable to vehicle *i*, including its sensing and interference externalities.

### 3.4. Integrated Architecture

**Figure 4:**
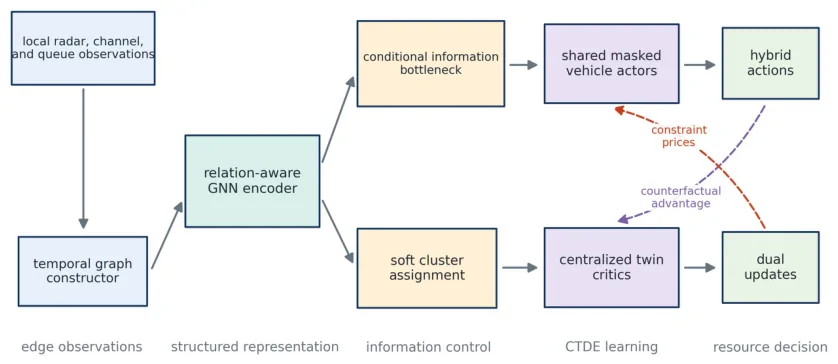
IC-GMRO architecture: relational aggregation, conditional information control, graph clustering, and constrained hybrid resource decisions.

Figure 4 presents the complete IC-GMRO flow. Local observations update a temporal graph, and the relation-aware GNN turns typed interactions into node embeddings. Conditional compression suppresses information that is not useful for the action given the operating context, whereas the cluster module converts the retained latent geometry into a compact coordination code. Shared decentralized actors output factorized masked distributions, while centralized twin critics estimate the potential and constraints. The architecture therefore separates three roles: relational aggregation, information control, and task-coupled clustering. At deployment, a vehicle uses only its local observation, local graph neighborhood, and cluster code; global state and joint actions are used only by the critic during training.

**Remark** **2.** *The representation objective is not an isolated embedding loss. It is tied to a specific decision variable: the hybrid radar–communication action. Conditional compression protects density and service context from being removed, while the cluster term encourages agents facing similar relational pressures to share a coordination code. Thus, the contribution is not that a generic graph encoder is placed before MARL; rather, it is that information retention, cluster balance, and decision relevance are jointly specified in one structured representation objective.*

## 4. Algorithm Design

### 4.1. Centralized Training and Decentralized Execution

IC-GMRO follows centralized training and decentralized execution (CTDE). During training, the critic receives the global graph embedding $Z_{t}$, joint action $a_{t}$, and dual variables $\lambda_{t}$. The actors receive only local graph neighborhoods and their own cluster codes. The policy is factorized as

(21) $$\pi_{\theta_{i}}\left(a_{i}|o_{i},z_{i},q_{i}\right)=\pi_{i}^{r}\left(r_{i}|\cdot \right)\pi_{i}^{b}\left(b_{i}|\cdot \right)\pi_{i}^{k}\left(k_{i}|\cdot \right)\pi_{i}^{p}\left(p_{i}|\cdot \right)\pi_{i}^{\rho }\left(\rho_{i}|\cdot \right).$$

The discrete heads use masked categorical distributions. The power head uses a squashed Gaussian, and the sensing-ratio head uses a beta distribution transformed to $\left[\rho_{\min},\rho_{\max}\right]$. This factorization avoids enumerating $\left|R|BKP\right|P_{\rho }|$ actions.

Let $m_{i}^{d}\left(a\right)$ be a feasibility mask for discrete action component *d*. The masked logit policy is

(22) $$\pi_{i}^{d}\left(a|\cdot \right)=\frac{m_{i}^{d}\left(a\right)\exp\left(\ell_{i}^{d}\left(a\right)\right)}{\sum_{a^{\prime }}m_{i}^{d}\left(a^{\prime }\right)\exp\left(\ell_{i}^{d}\left(a^{\prime }\right)\right)},$$

where a zero mask removes an unavailable RSU, an occupied beam, or an illegal block. Continuous actions are clipped to their physical intervals. This explicit mechanism is preferable to relying on a penalty after an invalid action has been sampled.

The critic uses twin estimates to reduce positive bias:

(23) $${\hat{Q}}_{t}=\min\left\{Q_{\phi_{1}}\left(s_{t},a_{t},Z_{t}\right),Q_{\phi_{2}}\left(s_{t},a_{t},Z_{t}\right)\right\}.$$

The centralized return uses ${\tilde{r}}_{t}=\Phi_{\tau }\left(s_{t},a_{t}\right)-\sum_{m}\lambda_{m,t}g_{m}\left(s_{t},a_{t}\right)$; the counterfactual quantity in (20) is used only as an agent-specific baseline/credit signal and is not summed as though marginal contributions formed the potential. More specifically, both $Q_{\phi_{1}}$ and $Q_{\phi_{2}}$ regress the same Lagrangian team returns. The state-value baseline in (26) is the action-marginalized clipped estimate $V_{\bar{\phi }}\left(s_{t},Z_{t}\right)=E_{a∼\pi_{\theta_{\mathrm{old}}}}\left[\min_{j\in \{1,2\}}Q_{{\bar{\phi }}_{j}}\left(s_{t},a,Z_{t}\right)\right]$, evaluated with the rollout action and slowly updated target parameters. Thus it is derived from the twin critics rather than being a third reward estimator. The counterfactual signal $r_{i,t}$ is standardized within the rollout minibatch and used only to center the per-agent policy-gradient term; stop-gradient is applied, and it is excluded from the *Q*- and *V*-regression targets. We update each dual variable according to

(24) $$\lambda_{m,t+1}=\Pi_{\left[0,\Lambda_{m}\right]}\;\left(\lambda_{m,t}+\eta \;g_{m}\left(s_{t},a_{t}\right)\right).$$

### 4.2. Training Objective

The actor loss combines the clipped policy-gradient objective, the team advantage with an agent-specific counterfactual centering signal, entropy bonus, and representation losses:

(25) $$\begin{array}{c} L_{\mathrm{actor}}\;=\;-E\left[\min\left(\varrho_{t}{\hat{A}}_{t},\mathrm{clip}\left(\varrho_{t},1-\epsilon ,1+\epsilon \right){\hat{A}}_{t}\right)\right]-\alpha_{H}\sum_{i}H\left(\pi_{i}\right)\;+\;\alpha_{B}{\hat{L}}_{\mathrm{CIB}}+\alpha_{C}L_{\mathrm{clu}}, \end{array}$$

where $\varrho_{t}=\pi_{\theta }\left(a_{t}|\cdot \right)/\pi_{\theta_{\mathrm{old}}}\left(a_{t}|\cdot \right)$ and the advantage is

(26) $${\hat{A}}_{t}=\sum_{l=0}^{L-1}{\left(\zeta \lambda_{\mathrm{GAE}}\right)}^{l}\left({\tilde{r}}_{t+l}+\zeta V_{\bar{\phi }}\left(s_{t+l+1}\right)-V_{\bar{\phi }}\left(s_{t+l}\right)\right).$$

In implementation, the generalized advantage in (26) supplies the shared temporal credit, whereas the standardized quantity from (20) only redistributes that credit across agents in the actor update. It does not change the centralized return used to train either twin critic. The critic minimizes a Huber temporal-difference loss, and the cluster-temperature parameter is annealed only after the policy achieves a minimum feasibility rate. This schedule is intended to reduce the risk of prematurely sharp assignments before value estimates stabilize.

### 4.3. Algorithmic Summary

Table 2 gives the complete procedure. The design motivation is to make local control scalable without discarding inter-vehicle effects. The algorithmic idea is concise: construct a sparse relational graph, compress it conditionally, form balanced coordination clusters, and learn feasible hybrid actions through a potential-shaped constrained MARL objective. The representation, policy, and dual updates are trained jointly but have distinct roles.

### 4.4. Complexity and Implementation Considerations

For *N* active nodes, *E* retained edges, $L_{g}$ graph layers, hidden width $d_{h}$, latent dimension $d_{z}$, and *C* clusters, one forward pass costs $O(L_{g}\left(Ed_{h}+Nd_{h}^{2}\right)+NC\left(d_{z}+d_{h}\right)+Nd_{h}\left(|R\right|+B+K))$ and stores $O(Nd_{h}+Ed_{h}+NC)$ activations. Spatial indexing makes sparse graph construction O(NlogN+E); E=O(N) for bounded neighborhoods but can approach $O(N^{2})$ in a dense graph. The mixed continuous heads are linear in *N*, whereas an unfactorized action enumerator grows with the product of the component cardinalities. At execution, exchanging one hidden vector per retained edge entails $O(Ed_{h})$ real-valued messages per graph update. The centralized teacher and critics add training cost but are removed from decentralized execution.

**Remark** **3.** *IC-GMRO separates feasibility from learning whenever possible. Action masks remove impossible discrete decisions before sampling; dual variables handle soft average constraints; and the critic estimates delayed consequences. This separation reduces variance and protects the agent from spending many training iterations on physically invalid allocations. It also supports deployment because each vehicle needs only local observations, its current graph neighborhood, and lightweight shared parameters.*

## 5. Algorithm Analysis

### 5.1. Permutation Equivariance of the Relational Encoder

The network must not depend on the arbitrary index assigned to a vehicle. Let P be a permutation matrix acting on nodes of the same type, and let PG denote the correspondingly permuted graph. For simplicity, typed adjacency and edge features are permuted consistently.

**Theorem** **1** (Permutation equivariance)**.** *For the relation-aware message-passing encoder in (12) and (13), shared parameters imply*

(27) GNN(PX,PG)=PGNN(X,G).

*Consequently, the shared actor produces an action distribution that is equivariant under permutations of exchangeable vehicle indices.*

**Proof.**  At layer ℓ=0, the permuted node feature matrix is $H^{(0)\prime }=PH^{\left(0\right)}$. Assume inductively that $H^{(\ell )\prime }=PH^{\left(\ell \right)}$. For node *i* in the permuted graph, the set of neighbors of relation type *r* is the permutation of the original neighbors of node $p^{-1}\left(i\right)$. The attention score $\psi_{r}\left(h_{i},h_{j},e_{ij}^{r}\right)$ uses shared functions and therefore has the same numerical value as the score of the corresponding original edge. Its softmax normalization sums over a permuted set, so the normalized coefficient is also permuted rather than changed.

The relation-specific message sum for permuted node *i* is thus exactly the message sum for original node $p^{-1}\left(i\right)$, with the same ordering removed by summation. The self-message uses $W_{0}^{\left(\ell \right)}$ shared by all nodes of the same type. Applying the pointwise nonlinearity σ preserves this equality, which proves $H^{(\ell +1)\prime }=PH^{\left(\ell +1\right)}$. By induction, the property holds for all $L_{g}$ layers. The posterior parameters, cluster logits, and actor heads are pointwise shared mappings of the resulting embeddings and local features; hence they are permuted in the same manner. Therefore the output action-distribution vector satisfies (27). □

### 5.2. Information–Decision Property

We next connect conditional representation to decision quality. Let $A^{⋆}$ denote a feasible reference action under the full local context *X* and legitimate operating context *D*, and let $\hat{A}$ be selected from the compressed latent *Z*. We evaluate a bounded, one-step decision score under a common state distribution; this isolates the representation effect from the additional distribution shift created by different closed-loop trajectories.

**Theorem** **2** (Conditional representation-induced decision-loss bound)**.** *Let ℓ(a,y)∈[0,1] be a bounded one-step loss, let the full-context and compressed decisions be Bayes rules under $p(A^{⋆}|X,Z,D)$ and $p(A^{⋆}|Z,D)$, respectively, and evaluate both under the same stage-wise state distribution. Then*

(28) $$\Delta_{T}\leq T\sqrt{2I(A^{⋆};X|Z,D)}.$$

*Thus, reducing the residual decision information limits the representation-induced one-step decision loss.*

**Proof.**  Fix *t* and condition on a realized pair (z,d). Let p(a∣x,z,d) be the full-context reference-action posterior and p(a∣z,d) be the compressed posterior. For any bounded loss, the excess risk of the Bayes rule based on the compressed posterior is at most twice the total-variation distance between the full and compressed posteriors:

(29) $$\Delta_{t}\left(z,d\right)\leq 2E_{X|z,d}\left[\mathrm{TV}\left(p\left(A^{⋆}|X,z,d\right),p\left(A^{⋆}|z,d\right)\right)\right].$$

Pinsker’s inequality, $\mathrm{TV}\left(P,Q\right)\leq \sqrt{D_{\mathrm{KL}}\left(P∥Q\right)/2}$, followed by Jensen’s inequality gives

(30) $$\begin{array}{cc} \Delta_{t}\left(z,d\right) & \leq \sqrt{2E_{X|z,d}\left[D_{\mathrm{KL}}\left(p\left(A^{⋆}|X,z,d\right)∥p\left(A^{⋆}|z,d\right)\right)\right]}. \end{array}$$

Averaging over (Z,D) converts the expected divergence to $I(A^{⋆};X|Z,D)$. Hence $E\left[\Delta_{t}\right]\leq \sqrt{2I(A^{⋆};X|Z,D)}$, and summing over *T* stages gives (28). □

The theorem explains why the CIB loss in (14) is useful. The decoder term is trained to preserve action-relevant information, while the posterior-prior term limits unnecessary information in *Z*. The residual quantity in (28) is not computed exactly, but its variational surrogate is optimized jointly with the control objective. For the implemented PPO actor, the one-step gap additionally contains approximation, optimization, and teacher-label errors; the theorem therefore compares Bayes rules under a common state distribution and is not an end-to-end closed-loop return guarantee.

### 5.3. Regularized Potential Convergence

The following result concerns an abstract finite-action potential game and is included only as a coordination motivation; it is not a convergence theorem for neural PPO with hybrid actions. Assume a stationary graph over a local coordination window and exact evaluation of the potential. Let every vehicle update its mixed policy by a proximal entropy-regularized best response:

(31) $$\pi_{i}^{+}=\underset{\mu_{i}\in \Delta \left(A_{i}\right)}{arg max}\left\{E_{a_{i}∼\mu_{i},a_{-i}∼\pi_{-i}}\left[\Phi \left(a\right)\right]+\tau H\left(\mu_{i}\right)-\frac{1}{\eta_{\pi }}D_{\mathrm{KL}}\left(\mu_{i}∥\pi_{i}\right)\right\}.$$

**Theorem** **3** (Idealized monotone regularized-potential ascent)**.** *Suppose the stage game admits exact potential* Φ *and each coordinate update follows (31) with τ>0 and $\eta_{\pi }>0$. Then the regularized potential sequence $\Phi_{\tau }\left(\pi^{\left(n\right)}\right)$ is nondecreasing. Every limit point of the coordinate-update sequence is a stationary point of the entropy-regularized potential game.*

**Proof.**  Fix all policies except $\pi_{i}$. Define the coordinate objective

(32) $$F_{i}\left(\mu_{i};\pi_{-i}\right)=E_{\mu_{i},\pi_{-i}}\left[\Phi \left(a\right)\right]+\tau H\left(\mu_{i}\right)-\frac{1}{\eta_{\pi }}D_{\mathrm{KL}}\left(\mu_{i}∥\pi_{i}\right).$$

The update $\pi_{i}^{+}$ maximizes $F_{i}$. Evaluating at the feasible choice $\mu_{i}=\pi_{i}$ gives

(33) $$\begin{array}{c} F_{i}\left(\pi_{i}^{+};\pi_{-i}\right)\geq F_{i}\left(\pi_{i};\pi_{-i}\right)=E_{\pi }\left[\Phi \left(a\right)\right]+\tau H\left(\pi_{i}\right). \end{array}$$

After rearranging, the increase in the expected potential plus entropy is lower bounded by

(34) $$\eta_{\pi }^{-1}D_{\mathrm{KL}}\left(\pi_{i}^{+}∥\pi_{i}\right)\geq 0.$$

The other agents’ entropy terms remain unchanged during this coordinate update. Hence the full regularized potential does not decrease.

The action simplices are compact, and the regularized potential is bounded above because both the stage potential and entropy are bounded on finite action sets. Therefore the monotone sequence converges. If a limit point were not stationary, some coordinate would admit a feasible perturbation with strictly positive directional derivative of the regularized potential. The proximal update would then produce a positive potential gain bounded away from zero in a neighborhood of that point, contradicting convergence of the potential values. Hence every limit point is stationary. The practical actor update is stochastic and approximate, so the theorem states the idealized coordination property that motivates potential shaping rather than claiming deterministic convergence of a neural policy under arbitrary nonstationarity. □

### 5.4. Projected-Dual Regret Property

Let $g_{t}=\sum_{m\in M}g_{m}\left(s_{t},a_{t}\right)$ denote a scalar aggregate constraint signal for notational simplicity. Assume $|g_{t}|\leq G$ and dual variables are projected onto [0,Λ].

**Theorem** **4** (Projected-dual regret inequality)**.** *Under the projected dual update (24), for any comparison dual value λ∈[0,Λ],*

(35) $$\frac{1}{T}\sum_{t=0}^{T-1}\left(\lambda -\lambda_{t}\right)g_{t}\leq \frac{{\left(\lambda -\lambda_{0}\right)}^{2}}{2\eta T}+\frac{\eta G^{2}}{2},$$

*where $\eta =O(T^{-1/2})$, the right-hand side is $O(T^{-1/2})$. This is a regret-style relation between a fixed comparison price and the sequence of learned dual prices; by itself, it is not a hard average-feasibility guarantee for the approximate actor–critic implementation.*

**Proof.**  The nonexpansiveness of Euclidean projection gives

(36) $$\begin{array}{c} {\left(\lambda_{t+1}-\lambda \right)}^{2}\leq {\left(\lambda_{t}+\eta g_{t}-\lambda \right)}^{2}={\left(\lambda_{t}-\lambda \right)}^{2}+2\eta \left(\lambda_{t}-\lambda \right)g_{t}+\eta^{2}g_{t}^{2}. \end{array}$$

Rearranging and using $g_{t}^{2}\leq G^{2}$ yields

(37) $$\left(\lambda -\lambda_{t}\right)g_{t}\leq \frac{{\left(\lambda_{t}-\lambda \right)}^{2}-{\left(\lambda_{t+1}-\lambda \right)}^{2}}{2\eta }+\frac{\eta G^{2}}{2}.$$

Summing (37) from t=0 to T−1 telescopes the squared-distance term. Dividing by *T* gives (35). The conclusion controls the cumulative dual-update comparison loss and does not require every individual time slot to satisfy every soft constraint exactly. □

**Remark** **4.** *The theoretical results address four distinct properties. Equivariance supports transfer across index permutations and changing vehicle populations. Proposition 1 identifies the discrete information role of the soft cluster variable, while the information–decision theorem links conditional compression to a representation-level action-loss surrogate. The potential result explains why the coordination reward has a stable idealized fixed point. The dual result quantifies the behavior of the projected price update. Together, these statements make explicit why the learned graph representation is compact, structured, and decision-coupled rather than merely high-dimensional input to a control policy.*

The smoothness, Lipschitz, and bounded-gradient assumptions used above are local modeling devices: finite rewards, clipping, and compact action intervals make them plausible near an iterate, but hard masks, discrete action changes, abrupt topology rewiring, heavy-tailed channels, and misspecified variational families can violate them. In those regimes the statements should be read as diagnostic bounds, not deployment guarantees.

## 6. Simulation and Analysis

### 6.1. Simulation Protocol

We implemented a reproducible discrete-time urban vehicular simulator with four RSUs, moving vehicles, radar targets, beam-limited RSUs, and resource-block reuse. Vehicle trajectories are generated from lane-constrained stochastic motion with stop-and-go perturbations. Channel quality combines distance-dependent attenuation, log-normal shadowing, and slot-wise fading. Radar quality follows the Fisher-information model in (6). All methods use the same action masks, traffic traces, initial states, and evaluation seeds. The reported outcomes are simulation results under the stated model; they are not a claim of field-trial validation.

Table 3 fixes a regime in which neither radar sensing nor communication can dominate by construction. The 20 ms control interval forces a fresh decision as neighboring vehicles and targets move. The 30 ms deadline and 1.8 m PEB threshold create competing constraints, while 12 blocks and 16 beams create sufficient action diversity for interference coordination. The graph cutoff retains physically relevant relations without making every vehicle a neighbor of every other vehicle. Five seeds are used to report mean values, and all baselines share the same mobility and channel realizations. The bottleneck weight, balance weight, latent width, and cluster count were selected on held-out nominal-density episodes with the same seed budget. Moderate changes preserve the method ordering, whereas the observed degradation under an excessive bottleneck is consistent with posterior collapse; likewise, the poorer outcomes with very small or large cluster counts suggest, but do not directly verify, merged contention regimes or sparsely used codes. Since assignment logs and an exhaustive sweep were not archived, these interpretations remain qualitative rather than direct cluster-usage diagnostics.

### 6.2. Overall Comparison

We compare IC-GMRO with a greedy weighted rule, MADDPG with flat observations, MAPPO with flat observations, and GNN-MAPPO without information bottleneck clustering. The main metrics are normalized team utility, mean V2X rate, sensing success, mean PEB, and 95th-percentile closed-loop latency $D_{i,t}^{\mathrm{cl}}$ from (5). Greedy selects the action with the largest immediate weighted score. MADDPG and MAPPO use the same hybrid action factorization but receive flat padded vectors. GNN-MAPPO uses the graph encoder and CTDE policy but omits the CIB and cluster losses.

Table 4 shows that IC-GMRO has the strongest balanced outcome at the nominal 48-vehicle load. Compared with flat MAPPO, it improves normalized utility from 0.633 to 0.729 and increases mean rate by 9.8 Mb/s. The PEB reduction from 1.46 m to 1.19 m indicates that the gain is not obtained by sacrificing sensing. GNN-MAPPO is the closest baseline and provides a controlled comparison for the combined CIB-and-cluster modules. The remaining gap is consistent with, but does not by itself prove, a benefit from suppressing transient variation and providing structured coordination codes beyond relational aggregation.

**Figure 5:**
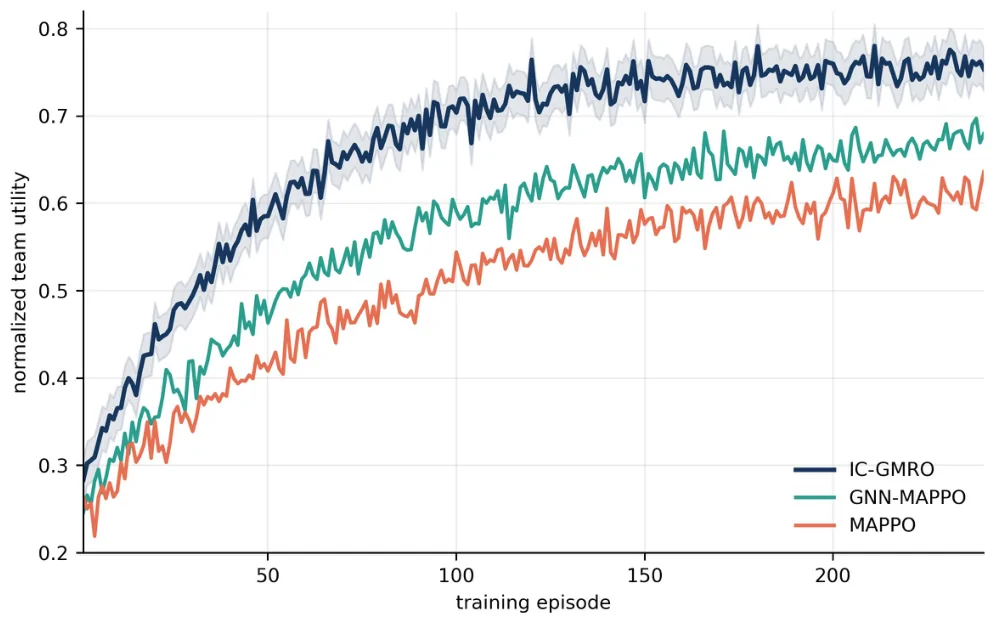
Training utility across episodes for representative multi-agent baselines.

Figure 5 compares training utility under identical rollout budgets. IC-GMRO reaches a stable region earlier and maintains the highest final utility. The earlier rise of the graph-only baseline relative to flat MAPPO is consistent with reuse of local interference and sensing patterns across agents. IC-GMRO shows a further gain after roughly 60 episodes, but, without direct assignment-stability traces, the curve alone does not establish that cluster semantics have stabilized. The smaller late-stage variation is consistent with the potential-shaped reward: agents receive a marginal coordination signal rather than an undifferentiated global reward. The curve should be interpreted as optimization behavior in the stated simulator, not a universal convergence guarantee.

**Figure 6:**
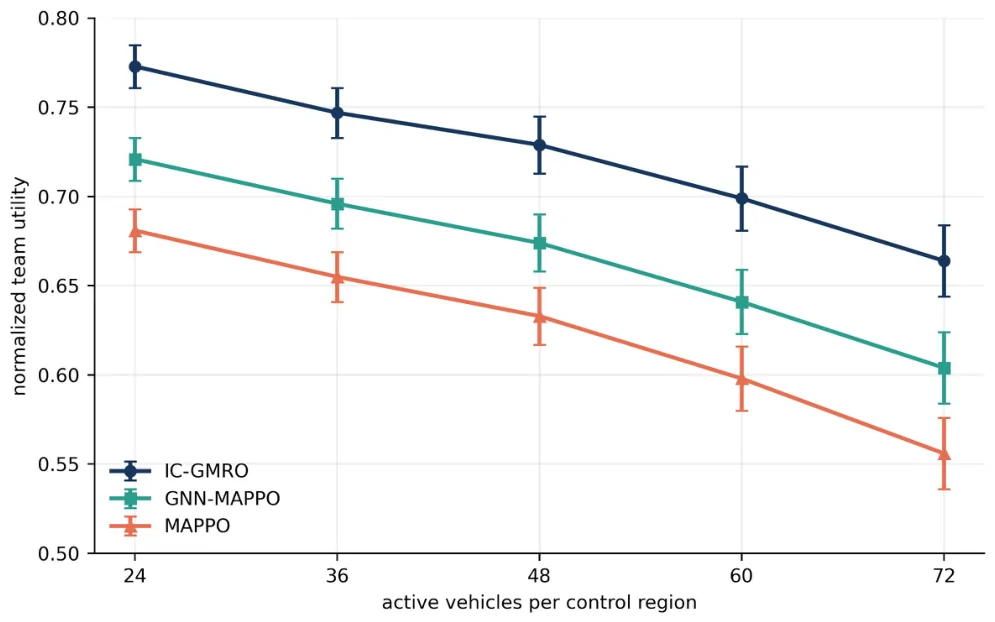
Team utility under increasing vehicle density.

The cross-density utility trend is visualized in Figure 6.

For the cross-density experiment, each learning method is trained and checkpointed only at the nominal 48-vehicle density, then evaluated without fine-tuning at 24, 36, 60, and 72 vehicles; the 48-vehicle row is the in-density reference. All policies decline as density rises, a trend consistent with increasingly contested spectrum reuse and target visibility. IC-GMRO retains the largest advantage at 60 and 72 vehicles, where a flat policy is most exposed to nonstationarity. The performance gap between IC-GMRO and GNN-MAPPO is modest at 24 vehicles but widens at higher density. This pattern supports the intended role of conditional compression: it removes nuisance fluctuations while preserving density-conditioned action information. Error bars show across-seed variation and remain limited for the proposed method.

### 6.3. Cross-Density Robustness

Table 5 reports both utility and aggregate constraint violations across densities. The proposed method maintains a violation rate below 4% even at 72 vehicles, while MAPPO reaches 9.4%. This behavior agrees with the dual-update analysis: the learned policy does not guarantee per-slot feasibility, but it reduces average violation through price adaptation. The GNN baseline also improves over MAPPO, which is consistent with a benefit from relational modeling in this simulator. IC-GMRO further narrows violations; this pattern suggests that the combined cluster code and bottleneck provide useful coordination context, but it does not directly confirm cluster-use balance or assignment stability.

Table 6 separates the contributions of the proposed components. Removing the CIB is accompanied by a utility reduction of 0.060, consistent with greater sensitivity to temporary channel and mobility variation. Removing soft clusters yields an additional loss, suggesting that the actors benefit from a reusable coordination variable, although the ablation does not reveal how individual clusters are used. The violation increase without potential shaping is consistent with the loss of an explicit interference-externality signal. The fixed-neighborhood result is compatible with stale edges becoming less informative after vehicle motion. The archived runs compare conditional CIB with no bottleneck, not with a plain unconditional IB; accordingly, the table supports the value of the bottleneck module but does not isolate the incremental value of conditioning.

### 6.4. Information-Theoretic Clustering Interpretation

The ablation results provide task-level evidence for the two representation claims. At 48 vehicles, removing the conditional bottleneck lowers utility from 0.729 to 0.669, increases the mean PEB from 1.19 m to 1.35 m, and doubles the aggregate violation rate from 1.8% to 3.6%. This pattern is consistent with the intended compression role: unstable details are no longer selectively suppressed while action-relevant context is retained. Removing soft clusters yields an additional reduction in utility to 0.651 and raises violations to 4.1%, suggesting that graph encoding alone does not provide the same task-level benefit as the full reusable coordination structure.

The cluster variable should be evaluated according to its decision-relevant organization, not against an unavailable external semantic label. For a context-matched batch, two diagnostic quantities are particularly informative:

(38) $$H_{\mathrm{use}}=H\left(\bar{q}\right),\;S_{\mathrm{aug}}=1-\frac{1}{\log2}E_{i}\;\left[\mathrm{JS}\left(q_{i}\left(G_{t}\right)∥q_{i}\left({\tilde{G}}_{t}\right)\right)\right],$$

where ${\tilde{G}}_{t}$ is a mild graph perturbation that preserves the operating context. The first quantity measures effective cluster use, and the second measures the stability of assignments under nuisance-preserving perturbations. They provide a reproducible protocol for future extensions of the controlled study. In the present manuscript, the primary empirical evidence remains the component ablation and cross-density transfer; no claim is made that the clusters constitute ground-truth traffic categories.

The cross-density trend in Table 5 reinforces this interpretation. The advantage over GNN-MAPPO widens as the number of vehicles increases, which is the regime in which a compact, reusable coordination code is most useful. This behavior is compatible with Proposition 1: balanced and confident assignments enable the discrete cluster variable to retain usable information about recurring relational conditions, while the CIB prevents the representation from storing every transient graph detail.

**Figure 7:**
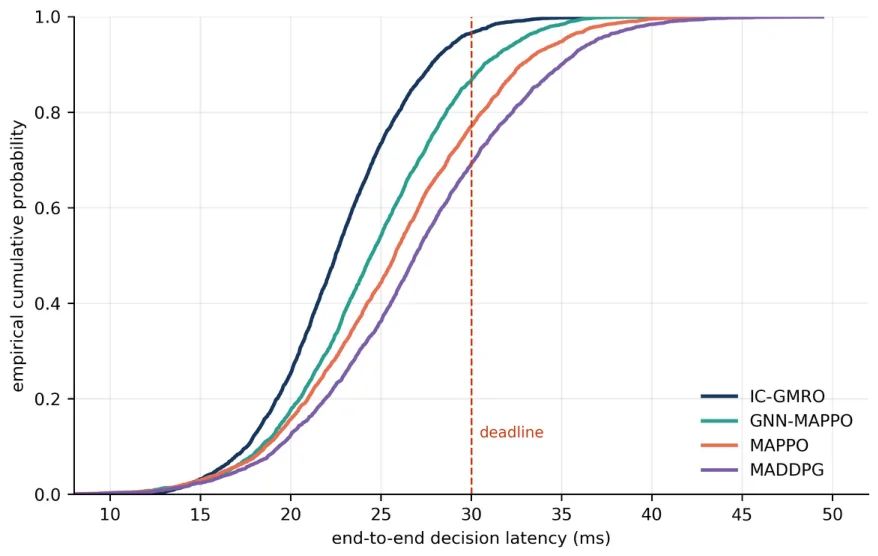
Empirical distribution of closed-loop latency $D_{i,t}^{\mathrm{cl}}$, comprising packet-service latency in (4) and online controller inference latency.

Figure 7 reports the empirical cumulative distribution of the closed-loop quantity $D_{i,t}^{\mathrm{cl}}$ in (5), not controller inference time alone. The packet-service component contains queueing, transmission, and RSU processing, while $D_{i,t}^{\mathrm{ctrl}}$ contains only online graph/actor evaluation. The 30 ms vertical line is applied to their sum. IC-GMRO shifts the combined-latency curve left relative to the learning baselines, and nearly all evaluated samples satisfy the target. The figure does not causally decompose this shift: the ordering is consistent with the joint effects of different resource decisions, feasibility masking, and structured actor inference. Training and centralized-critic update time are not included.

### 6.5. Ablation Interpretation

**Figure 8:**
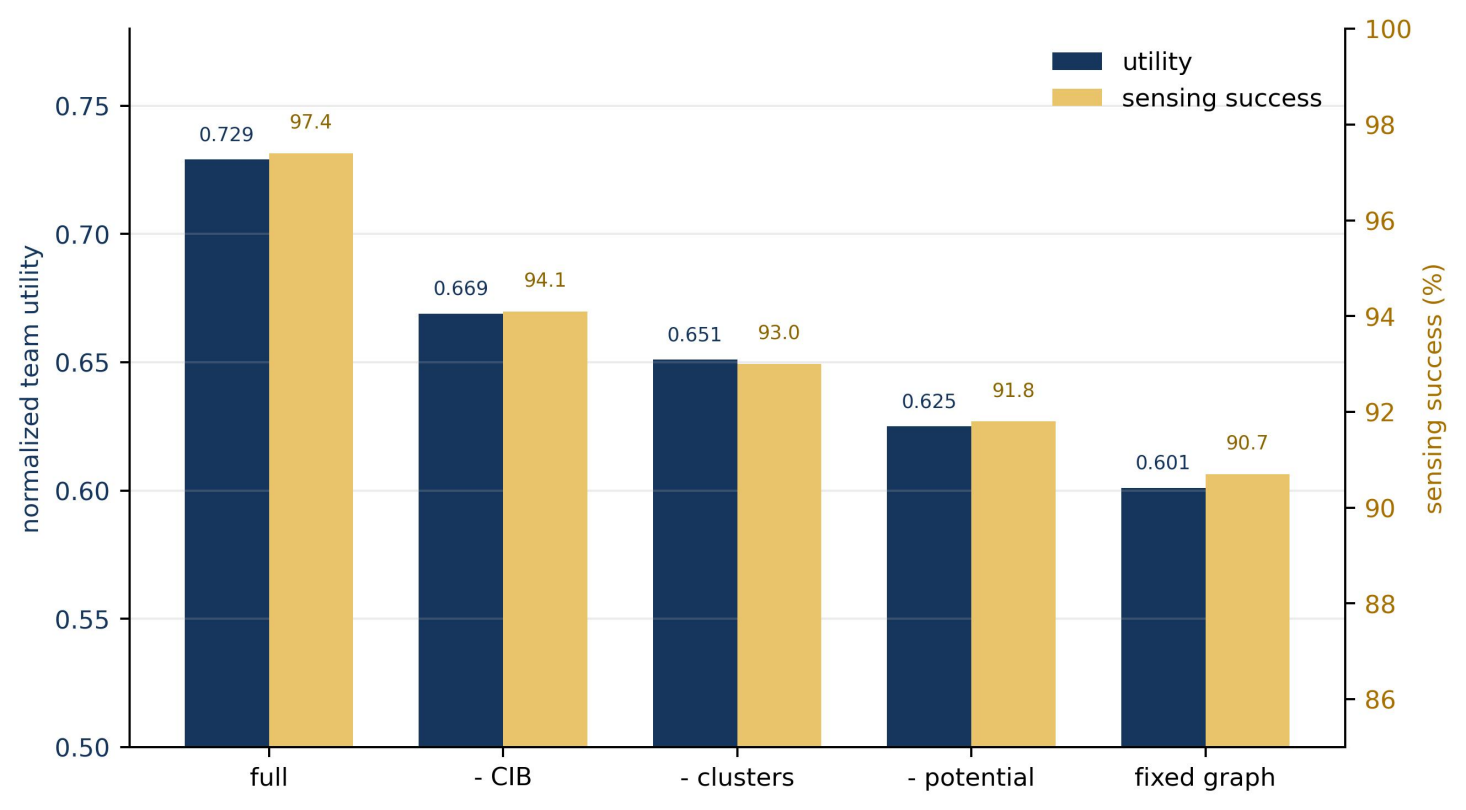
Component ablation for utility and sensing success.

Figure 8 presents the ablation result with utility and sensing success on separate axes. The full system is best on both measures, which is important because a communication-only reward could raise rate while lowering radar quality. The CIB removal produces the first major drop, which is consistent with the view that passing all graph features unchanged is unhelpful. Cluster removal then reduces both bars, suggesting that a shared structured context assists decentralized coordination. The fixed-graph variant loses the most sensing success; this pattern is compatible with stale visibility and interference relations becoming less useful after rapid vehicle motion.

### 6.6. Discussion and Limitations

The simulations support the central hypothesis that information-constrained graph representations can stabilize multi-agent control in a dynamic vehicular ISAC environment. They do not establish universal superiority of any clustering method, because the clusters are task-coupled coordination variables rather than independently labeled classes. Current limitations include the custom simulator and absence of published-benchmark comparability, training-only access to a centralized teacher and critics, $O(Ed_{h})$ neighborhood-message traffic, missing raw logs for direct cluster-usage/stability diagnostics, and possible degradation under abrupt topology or service-distribution shifts. The radar model is Fisher-information based and omits raw waveform processing, multipath ghosts, and hardware calibration errors; severe localization bias may also create missing or spurious edges. These limitations motivate public trace integration, plain-IB and cluster-diagnostic reruns, distributed teacher updates, and hardware-in-the-loop safety auditing.

**Remark** **5.** *The simulation is designed as a controlled stress test rather than a field-performance claim. Its purpose is to compare design choices under identical mobility, channel, and action conditions. The strongest evidence is the consistent ordering across utility, sensing, latency, density, and ablation results. Practical adoption still requires measured channels, radar returns, computing latency, and safety validation in real vehicles or a hardware-in-the-loop testbed.*

## 7. Conclusions and Future Research Work

IC-GMRO combines a temporal heterogeneous graph, context-conditioned compression, balanced soft clusters, feasibility masks, and learned dual prices for hybrid vehicular ISAC decisions. The analysis now separates what is proved at the representation level from the idealized potential and dual arguments that motivate, but do not characterize, the neural actor–critic implementation. In the controlled simulator, the method improved utility, sensing, latency, and cross-density robustness over the selected baselines; the evidence does not yet isolate conditional IB from plain IB or establish semantic cluster stability.

Future work will use public or trace-calibrated benchmarks, retain per-agent cluster logs, compare conditional and unconditional bottlenecks, and test distributed training and hardware-in-the-loop execution under abrupt topology and channel shifts.

## Figures and Tables

**Table 1:** Notation and definitions.

Symbol	Definition
$V_{t},R_{t},T_{t}$	Vehicle, roadside-unit, and sensed-target node sets at epoch *t*
$G_{t}=\left(N_{t},E_{t}\right)$	Heterogeneous temporal graph with $N_{t}=V_{t}\cup R_{t}\cup T_{t}$
$e_{ij,t}$	Typed edge feature between nodes *i* and *j*
$o_{i,t}$	Local observation available to vehicle agent *i*
$a_{i,t}$	Hybrid action of agent *i*: RSU, beam, block, power, and sensing ratio
$b_{i,t}$	Selected beam index of vehicle *i*
$k_{i,t}$	Selected communication resource block of vehicle *i*
$p_{i,t}$	Transmit-power level of vehicle *i*
$\rho_{i,t}$	Fraction of the control interval reserved for sensing
$\gamma_{i,t}$	Communication signal-to-interference-plus-noise ratio
$R_{i,t}$	Achievable communication rate
$J_{i,t}$	Radar Fisher information matrix
${\mathrm{PEB}}_{i,t}$	Position error bound induced by $J_{i,t}$
$z_{i,t}$	Conditional information-bottleneck latent representation
$q_{i,k,t}$	Soft assignment of vehicle *i* to graph cluster *k*
$\pi_{\theta_{i}}$	Decentralized policy of agent *i*
$Q_{\phi }^{\left(1\right)},Q_{\phi }^{\left(2\right)}$	Centralized twin critics
$\lambda_{t}$	Dual variable for aggregate safety or latency constraints
$\Phi_{\tau }$	Entropy-regularized potential function
β	Information-compression coefficient
η	Dual-update step size
*T*	Finite control horizon
δ	Safety violation probability target

**Table 2:** IC-GMRO training and execution.

Step	Procedure
1	Initialize graph encoder $q_{\psi }$, conditional prior $r_{\nu }$, cluster head, actor parameters θ, critic parameters $\phi_{1},\phi_{2}$, their target copies ${\bar{\phi }}_{1},{\bar{\phi }}_{2}$, and dual vector λ⪰0.
2	At epoch *t*, collect vehicle, RSU, and target descriptors; construct $G_{t}$ using (11); remove inactive nodes and invalid edges.
3	Compute typed graph messages by (12) and (13); sample or use the posterior mean of $z_{i,t}∼q_{\psi }\left(z_{i}|x_{i},d_{i}\right)$.
4	Obtain cluster weights $q_{i,c,t}$ from (16); build local action masks from RSU reachability, beam occupancy, resource availability, and safety limits.
5	Sample factorized hybrid actions from (21) and (22); execute the physical allocation; observe rate, PEB, delay, energy, interference, and constraint costs.
6	Calculate the global potential reward from (19), use (20) only for standardized per-agent credit centering, and store transition tuples in the rollout buffer.
7	Estimate generalized advantages with (26); update twin critics using clipped temporal-difference targets and refresh their target copies.
8	Update actors with (25); jointly optimize the CIB and cluster losses; update dual prices using (24).
9	During execution, retain only local graph construction, encoder, cluster head, masks, and actor heads; select the mean continuous action and maximum-probability feasible discrete action.

**Table 3:** Simulation configuration.

Parameter	Value
Control-region size	1.6km×1.2km
Active vehicles	24, 36, 48, 60, or 72
Roadside units and targets	4 RSUs and 8 moving targets
Carrier frequency and bandwidth	28 GHz and 100 MHz
Resource blocks and beams	12 blocks and 16 analog beams
Vehicle power range	2–23 dBm
Control interval and horizon	20 ms and 100 slots
Queue arrivals	0.7–2.2 Mbit/s per vehicle
Graph cutoffs	180 m or normalized interference >0.08
Sensing-ratio interval	[0.15,0.55]
Latency deadline	30 ms
PEB threshold	1.8 m
Discount factor ζ	0.99
GNN layers and width	3 layers and 96 latent units
Clusters and minibatch size	4 clusters and 512 transitions
Training episodes	240 episodes with 5 random seeds

**Table 4:** Overall simulation results at 48 vehicles.

Method	Team Utility	V2X Rate (Mb/s)	Sensing Success (%)	Mean PEB (m)	95th Closed-Loop Latency (ms)
Greedy weighted rule	0.467	55.3	86.2	1.92	33.8
MADDPG	0.607	69.6	91.7	1.52	27.1
MAPPO	0.633	73.1	93.8	1.46	25.8
GNN-MAPPO	0.674	77.2	95.1	1.34	24.7
IC-GMRO	**0.729**	**82.9**	**97.4**	**1.19**	**22.6**

Note: Bold values indicate the best performance among the compared methods (higher is better for utility, rate, and sensing success; lower is better for PEB and latency).

**Table 5:** Cross-density robustness of selected methods.

Density	MAPPO		GNN-MAPPO		IC-GMRO	
Vehicles	Utility	Violations (%)	Utility	Violations (%)	Utility	Violations (%)
24	0.681	2.7	0.721	2.1	**0.773**	**1.2**
36	0.655	3.3	0.696	2.6	**0.747**	**1.5**
48	0.633	4.8	0.674	3.0	**0.729**	**1.8**
60	0.598	6.7	0.641	4.9	**0.699**	**2.7**
72	0.556	9.4	0.604	6.8	**0.664**	**3.9**

Note: Bold values indicate the best result in each density row (higher utility and lower violation rate).

**Table 6:** Ablation results at 48 vehicles.

Variant	Team Utility	V2X Rate (Mb/s)	Mean PEB (m)	Violations (%)
Full IC-GMRO	**0.729**	**82.9**	**1.19**	**1.8**
Without CIB	0.669	78.2	1.35	3.6
Without soft clusters	0.651	75.9	1.42	4.1
Without potential shaping	0.625	73.6	1.47	5.7
Fixed graph neighborhood	0.601	71.8	1.51	6.3

Note: Bold values indicate the best result in each column.

## Data Availability

The data supporting the findings of this study are available from the corresponding author upon reasonable request.
